# From neglected to notable: A growing public health challenge driven by hospitalization for sporotrichosis in Pernambuco, Northeast Brazil (2016–2024)

**DOI:** 10.1371/journal.pntd.0013795

**Published:** 2025-12-01

**Authors:** Filipe Prohaska-Batista, Francelise Bridi Cavassin, Flavio de Queiroz-Telles

**Affiliations:** 1 Hospital Universitário Oswaldo Cruz (HUOC), Recife, Pernambuco, Brasil; 2 Faculdades Pequeno Príncipe (FPP), Curitiba, Paraná, Brasil; 3 Departamento de Saúde Coletiva, Universidade Federal do Paraná (UFPR), Curitiba, Paraná, Brasil; National Institute for Communicable Diseases, Johannesburg, South Africa, SOUTH AFRICA

## Abstract

Opportunistic and endemic mycoses represent a significant global health concern, particularly in Brazil. In the northeastern state of Pernambuco (PE), sporotrichosis has become a rapidly escalating public health challenge, evidenced by a notable increase in hospital admissions, despite its mostly outpatient nature. The present study, therefore, aimed to investigate the epidemiological and clinical profile of mycosis-related hospitalizations in PE from 2016 to 2024, with a primary focus on the burden of sporotrichosis. This statewide surveillance study used data from Brazil’s Unified Health System Hospital Information System (SIH/SUS) to conduct a descriptive and comparative assessment of hospitalizations for ten fungal diseases in PE and nationally. Incidence rates and the Error, Trend, Seasonality (ETS) framework were applied to forecast case numbers for 2025. A total of 794 hospitalizations were recorded in PE during the study period. The most prevalent infections were aspergillosis (23.0%), sporotrichosis (17.5%), cryptococcosis (15.1%), and chromoblastomycosis (13.6%), with an overall mortality rate of 10.5%. Hospitalizations for sporotrichosis rose from five cases in 2016–28 in 2021, with local incidence rates surpassing the national average. This implantation mycosis showed a marked concentration in Recife, reflecting the zoonotic spread of *Sporothrix brasiliensis* in urban areas. Notably, 46.0% of sporotrichosis cases were classified as unspecified, underscoring gaps in reporting. Among specified cases, the lymphocutaneous form predominated (18.7%), followed by disseminated disease (17.3%). Forecasts for 2025 indicate a persistently high hospitalization burden. These findings highlight a pressing public health issue in PE. The surge in sporotrichosis cases and the high share of severe and unspecified forms point to weaknesses in diagnosis and data recording. Tackling this emerging endemic-epidemic threat requires improved surveillance, mandatory notification, and better data capture in SIH/SUS, alongside integrated actions across human, animal, and environmental health sectors.

## Author summary

Fungal infections represent a serious and often neglected global health threat, demanding effective surveillance, especially in endemic areas and tropical climates. Our study focuses on this burden in Pernambuco (PE), a northeastern Brazilian state. While severe opportunistic infections usually dominate hospital admissions, our analysis over nine years reveals a surprising epidemiological shift: sporotrichosis, a mycosis traditionally treated outside hospitals, has emerged as the second most common cause of fungal-related hospitalizations in the area. Local sporotrichosis rates in PE now notably exceed the national average. This spike results from the ongoing epidemic of zoonotic transmission from domestic cats, a trend that may have been worsened by increased pet adoption during COVID-19 lockdowns. We also observed severe (disseminated) and unclassified cases, indicating significant gaps in early diagnosis and official reporting across the region. This study highlights an urgent, emerging public health crisis in PE. To effectively address this endemic-epidemic issue, our findings recommend immediate measures, including enhanced mandatory notification, improved data collection in surveillance systems, and the adoption of integrated One Health policies across the human, animal, and environmental health sectors.

## Introduction

Opportunistic and endemic mycoses impose a growing global burden, with millions of acute cases and annual deaths [1]. The increasing population of immunocompromised individuals drives this scenario, as well as the expansion of invasive procedures and shortcomings in diagnostic methods and antifungal availability [2].

In Brazil, where the tropical climate and socioeconomic disparities favor fungal proliferation, over 3.8 million people develop severe fungal infections each year, compounded by the lack of vaccines and effective prophylaxis on a large scale [3]. The public health burden of mycoses in Brazil is complex, driven by both primary endemic mycoses (paracoccidioidomycosis, histoplasmosis, and chromoblastomycosis) and life-threatening opportunistic mycoses (including cryptococcosis, aspergillosis, and invasive candidiasis) that mostly affect immunocompromised populations. The clinical and hospital impacts of these mycoses are significant. Such hospitalizations require prolonged and expensive treatments, in addition to straining intensive care units and highlighting the need for early diagnosis and effective antifungal therapies.

Implantation mycoses, resulting from traumatic inoculation, also account for a huge portion of these diseases, notably sporotrichosis caused by *Sporothrix brasiliensis*, which is endemic across 26 of Brazil’s 27 federative units [4]. Sporotrichosis is included among the fungal skin neglected tropical diseases (NTDs) that are recognized by the World Health Organization (WHO) [5]. This classification is due to the significant morbidity this disease causes worldwide, as well as its potential for geographic expansion related to climate change and socioeconomic impact, particularly in low-income countries [6,7]. There has been a notable increase in incidence, specifically in urban areas, due to zoonotic transmission from infected cats. This trend has been reported in various regions of the country [8–12].

Between 1992 and 2015, 782 hospitalizations for sporotrichosis were recorded in Brazil [13]. Another survey counted 672 hospitalizations from 2007 to 2018 (an average of 56 per year), accentuating excessive costs and the severity of extracutaneous forms, potentially fatal in immunocompromised patients, leading to the disease’s mandatory notification in January 2025 [14,15].

Pernambuco (PE), in Northeastern Brazil, presents a unique scenario: its dense urban centers, coastal climate, and socioeconomic disparities create conditions that are both conducive to fungal proliferation and gaps in healthcare access. The geographic concentration of human cases in Olinda, Jaboatão dos Guararapes, and Recife forms a recognizable cluster that strongly indicates active zoonotic transmission, associated with the spread of S. *brasiliensis* by domestic cats in these urban areas [16,17]. However, given the mostly outpatient nature of this implantation mycosis, few studies have systematically assessed the hospital burden in this region.

Understanding the temporal and geographic patterns of these infections is essential for informing control strategies, guiding resource allocation, and anticipating emerging hotspots. This study analyzes nine years of hospital admission data for sporotrichosis and other opportunistic and endemic fungal diseases in PE, applying time-series modeling and geographic analysis to identify trends and explore their implications within a One Health perspective.

## Methods

### Ethics statement

This study used publicly available, de-identified secondary data, exempting it from institutional ethics review in accordance with Brazilian Resolution CNS 510/2016.

### Study design and data source

A statewide surveillance study was conducted on hospitalized patients diagnosed with opportunistic and endemic mycoses over nine years, from 2016 to 2024. The data was obtained from the public domain nationwide database of the Hospital Information System of the Unified Health System (SIH/SUS), provided by the Brazilian Ministry of Health (MoH). SIH/SUS is a clinical and administrative database that stores data on hospital admissions nationwide. This system is used to monitor and evaluate the performance of hospitals, both public and private, that provide services to the SUS. SIH/SUS is also used to validate and record hospitalizations for conditions sensitive to primary care, such as infectious diseases, which helps identify areas where primary care can be strengthened to reduce unnecessary hospitalizations.

Mycoses were classified according to the International Classification of Diseases (ICD-10), which is a standard classification used globally to record and monitor diseases and health conditions. Data on hospital admissions for fungal infections in PE, registered in the SIH/SUS, were obtained using the related codes and their subcategories. Sporotrichosis cases were identified under the primary ICD-10 code B42.

Admissions were defined as patients hospitalized for a mycosis, meaning the fungal disease was recorded as the principal diagnosis according to the SIH/SUS classification. To quantify the prevalence of hospitalized patients, the data were filtered to include only one unique hospitalization event per patient per specific mycosis during the study period (2016–2024). This approach ensured that a single patient was counted only once for a given fungal infection, even if they had multiple admission episodes recorded in the SIH/SUS system.

Although detailed clinical comorbidity data are not available within the SIH/SUS database, we recognize the need to contextualize the population at risk for invasive mycoses. To this end, we note that the state of PE presents a significant burden of immunosuppression. According to the most recent data (referring to 2023), the AIDS detection rate in the PE state was 16.9 cases per 100,000 inhabitants. In the capital, Recife, this rate rises to 26.4 cases per 100,000 inhabitants.

The concentration of mycosis-related hospital cases in Recife is a direct reflection of the region’s healthcare infrastructure. PE concentrates its high-complexity services in Recife, including teaching and reference hospitals. These units serve as reference centers for specialties such as hematology, oncology, transplantation, and the treatment of severe infectious diseases. They are the primary receiving facilities for patients with invasive mycoses.

Mortality was defined as all-cause in-hospital death recorded as the outcome of the admission episode in the SIH/SUS database. Due to data limitations within the available system, it was not feasible to calculate mycosis-specific case-fatality proportions or determine whether death was directly attributable to the fungal infection.

### Statistical analysis

The study performed a descriptive and comparative analysis of the incidence of ten fungal diseases, focusing on the state of PE versus the national aggregate of Brazil. Case counts were obtained from the SIH/SUS, and population data for each federative unit and for Brazil were retrieved programmatically in R [18] via the ‘sidrar’ package from the 2022 IBGE Demographic Census [19]. Incidence rates per 1,000 inhabitants were calculated for each disease as (number of cases/ total population) × 1,000, separately for PE and for Brazil, and the disparity between them was expressed as (rate_PE/ rate_Brazil) × 100%.

All counts, population figures, incidence rates, and percentage ratios were compiled into descriptive tables. Comparative graphs were created side by side for PE and Brazil using ‘dplyr’ for data manipulation, ‘ggplot2’ for plotting, and scales for formatting, with numeric annotations displayed on each point to aid interpretation. To forecast the number of cases in 2025, annual case counts from 2016 to 2024 were modeled using the ETS (Error, Trend, Seasonality) framework via the forecast package in R, selecting the ETS (M, N, N) configuration by minimizing the corrected Akaike Information Criterion (AICc). This model, which incorporates multiplicative errors without trend or seasonal components, produced point forecasts and 95% prediction intervals for 2025.

Quantitative variables underwent Shapiro–Wilk testing for normality; normally distributed data are reported as mean ± standard deviation, while non-normal data are presented as median (minimum–maximum). Qualitative variables are expressed as absolute counts and percentages. Statistical significance between two groups was assessed using Student’s t-test for standard quantitative variables, the Mann–Whitney U test for non-normal variables, and Pearson’s chi-square test with continuity correction for categorical comparisons. All statistical analyses, tables, and figures were generated in Jamovi version 2.5.0 (based on R) [20] and in R version 4.2.1 [18].

## Results

The data on all mycoses are presented to establish the necessary public health context, considering the historically prevalent burden of opportunistic infections in PE hospital admissions. This comprehensive view is essential for accurately framing the epidemiological impact of sporotrichosis, which has emerged as the primary mycosis of concern during the study period.

### Exploration of demographic and clinical attributes of patients affected by opportunistic and endemic mycoses, including sporotrichosis, in Pernambuco, Brazil

The sociodemographic and clinical profile of 794 hospitalizations for opportunistic and endemic mycoses recorded in PE between 2016 and 2024 is presented (Table 1). During the studied period, ten different mycoses resulting in hospital admissions were reported. Of the total patients, 57.3% were male and 42.7% were female; their ages ranged from newborn to 102 years, with a median age of 47 years (0–102). Among the mycoses resulting in hospital admissions, the most prevalent were aspergillosis (23.0%), sporotrichosis (17.5%), cryptococcosis (15.1%), and chromoblastomycosis (13.6%). Clinical discharge was observed in 89.5% of cases, whereas 10.5% progressed to a fatal outcome.

**Table 1:** Demographic and clinical-epidemiological characteristics of mycosis-related hospitalizations in Pernambuco, Brazil (2016–2024).

Variables	Total	%
*Hospital admissions (N)*	794	100
*Sex*		
Female	339	42.7
Male	455	57.3
*Age group (years)*		
0 - 17	137	17.3
18 - 102	657	82.7
Median age, years [IQR]	47 [0-102]	
*Ethnicity*		
White	75	9.4
Black	20	2.5
East Asian	13	1.6
Mixed-race	530	66.8
Indigenous	1	0.1
Not informed	155	19.5
*Year of hospitalization*		
2016	65	8.2
2017	75	9.4
2018	113	14.2
2019	103	13.0
2020	112	14.1
2021	123	15.5
2022	83	10.5
2023	53	6.7
2024	67	8.4
*Fungal infection based on ICD-10*		
Aspergillosis (B44.0)	183	23.0
Invasive Candidiasis/Candidemia (B37.7)	84	10.6
Coccidioidomycosis (B38)	46	5.8
Cryptococcosis (B45)	120	15.1
Chromoblastomycosis (B43)	108	13.6
Sporotrichosis (B42)	139	17.5
Eumycetoma (B47.1)	4	0.5
Histoplasmosis (B39)	50	6.3
Mucormycosis (B46)	10	1.3
Paracoccidioidomycosis (B41)	50	6.3
*Outcomes*		
Improved hospital discharge	711	89.5
Death	83	10.5

Legend: N, number of patients, ICD, International Classification of Diseases.

Table 2 presents a comprehensive profile of the 139 hospital admissions attributed to sporotrichosis, accounting for 17.5% of all mycosis-related hospitalizations. The sex distribution was balanced (48.2% female vs. 51.8% male), with a median age of 49 years (range: 0–92). The municipalities where cases were reported are also listed, with Recife showing the highest number of cases (77.7%), followed by Paulista (6.5%) and Sirinhaem (4.3%). Admissions rose from 5 in 2016–28 in 2021, then dipped in 2022–2023, before reaching 13 in 2024. Clinical forms were unspecified in 46.0% of cases. Of the specified forms of sporotrichosis, the lymphocutaneous form predominated (18.7%), followed by disseminated (17.3%), extracutaneous (13.7%), and pulmonary (4.3%) infection. Regarding hospitalization outcomes, 77.7% were discharged, 5.8% resulted in death, and 16.5% had no recorded outcome.

**Table 2:** Epidemiological overview of sporotrichosis hospitalizations in Pernambuco, Brazil (2016–2024).

Variables	Total	%
*Hospital admissions (N)*	139	100
*Sex*		
Female	67	48.2
Male	72	51.8
*Age group (years)*		
0 - 17	20	14.4
18 - 92	119	85.6
Median age (years) [IQR]	49 [0-92]	
*Ethnicity*		
White	11	7.9
Black	2	1.4
East Asian	3	2.2
Mixed-race	90	64.7
Not informed	33	23.7
*Municipalities*		
Araripina	3	2.2
Barreiros	1	0.7
Bezerros	1	0.7
Cabrobo	1	0.7
Carpina	1	0.7
Caruaru	3	2.2
Feira Nova	1	0.7
Jaboatão dos Guararapes	1	0.7
Mirandiba	1	0.7
Ouricuri	1	0.7
Paulista	9	6.5
Recife	108	77.7
Sertania	1	0.7
Sirinhaem	6	4.3
Surubim	1	0.7
*Year of hospitalization*		
2016	5	3.6
2017	6	4.3
2018	12	8.6
2019	20	14.4
2020	21	15.1
2021	28	20.1
2022	23	16.5
2023	11	7.9
2024	13	9.4
*Clinical forms Sporotrichosis (ICD-10-B42)*		
Pulmonary (B42.0)	6	4.3
Lymphocutaneous (B42.1)	26	18.7
Disseminated (B42.7)	24	17.3
Other forms (B42.8)	19	13.7
Non-specified (B42.9)	64	46.0
*Outcomes*		
Improved hospital discharge	108	77.7
Death	8	5.8
Not informed	23	16.5

Legend: N, number of patients; ICD, International Classification of Diseases.

### Hospital admissions incidence rates

**Fig 1:**
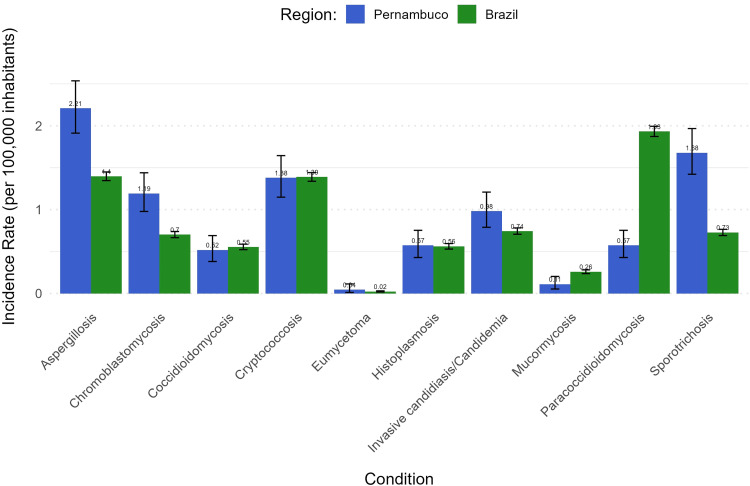
Hospital incidence rates of mycoses in Pernambuco vs. Brazil (per 1,000 inhabitants), including 95% Confidence Intervals (95% CI).

Annual hospital admissions incidence rates (per 1,000 inhabitants) for opportunistic and endemic mycoses were compared between PE and the national average (Fig 1). Aspergillosis had the highest rates in both contexts, followed by cryptococcosis, invasive candidiasis, and histoplasmosis. PE showed higher rates for sporotrichosis and chromoblastomycosis, indicating a disproportionately high local burden. Although sporotrichosis was less common than aspergillosis, its local rate exceeded the national level.

### Geographic distribution of cases of opportunistic and endemic mycoses, including sporotrichosis in Pernambuco, Brazil

**Fig 2:**
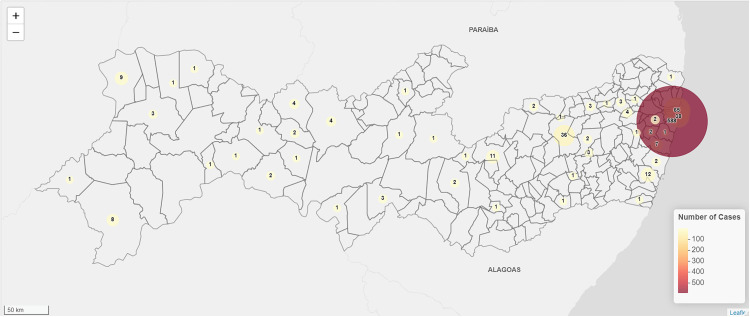
Geographic distribution of hospitalizations for opportunistic and endemic mycoses in Pernambuco, Brazil (2016-2024) [18,19]. The data is accessed programmatically via the geobr R package, which sources its data directly from IBGE. A representative link to the source data is: https://www.ibge.gov.br/geociencias/organizacao-do-territorio/malhas-territoriais.html.

**Fig 3:**
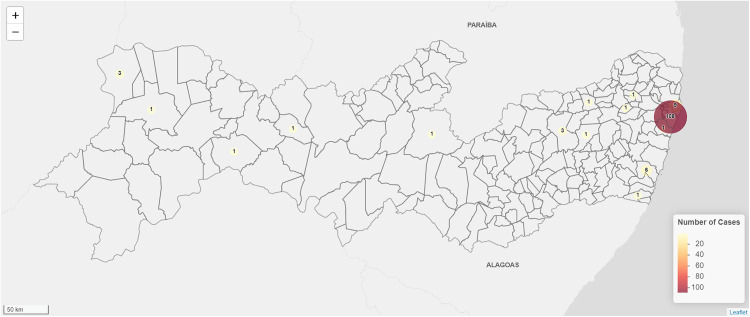
Geographic distribution of sporotrichosis hospitalizations in Pernambuco, Brazil (2016-2024) [18,19]. The data is accessed programmatically via the geobr R package, which sources its data directly from IBGE. A representative link to the source data is: https://www.ibge.gov.br/geociencias/organizacao-do-territorio/malhas-territoriais.html.

The geographic distribution of the 794 hospitalizations for opportunistic and endemic mycoses is depicted on a choropleth map (Fig 2). A clear hotspot is evident in Recife, with the highest densities observed in Recife, Jaboatão dos Guararapes, and Olinda. Conversely, interior regions recorded a significantly lower number (≤5) of admissions over the nine years. Fig 3 focuses on 139 admissions for sporotrichosis. Recife leads with 108 cases (77.7%), followed by Paulista (6.5%) and Sirinhaém (4.3%). Caruaru and Araripina each reported three cases (2.2%), and 11 other municipalities reported one case each (0.7%).

These demographic patterns align with the geographic concentration of cases, particularly in Recife, suggesting a link between urban density and hospitalization risk.

### Clinical manifestations of hospitalized patients with sporotrichosis

**Fig 4:**
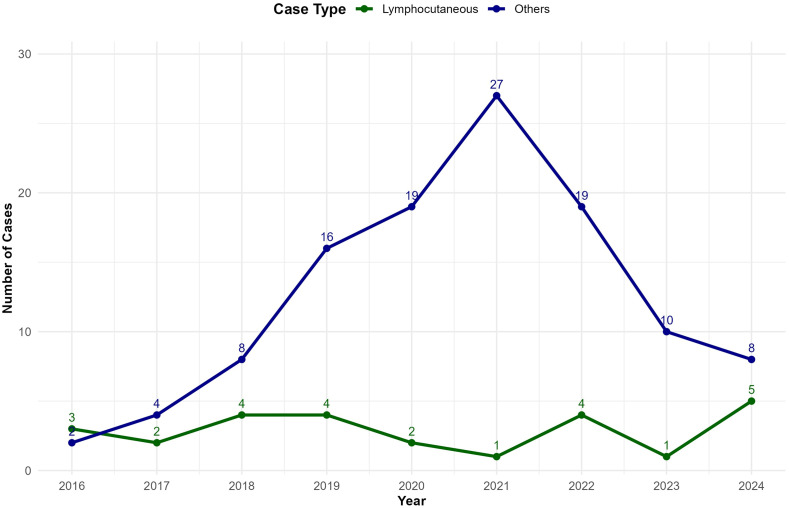
Clinical manifestations of hospitalized patients with sporotrichosis (2016–2024).

Fig 4 displays the relative frequency of five clinical categories among 139 hospitalizations for sporotrichosis. Unspecified presentations (B42.9) accounted for 46%, indicating significant gaps in diagnostic reporting. Among specified cases, lymphocutaneous (B42.1) was the most common (18.7%), followed by disseminated (B42.7; 17.3%), extracutaneous/other (B42.8; 13.7%), and pulmonary (B42.0; 4.3%). While the lymphocutaneous form remains predominant, the substantial share of systemic presentations displays the need for rigorous classification protocols and prompt identification of severe forms.

### Hospital admissions estimate for 2025

**Fig 5:**
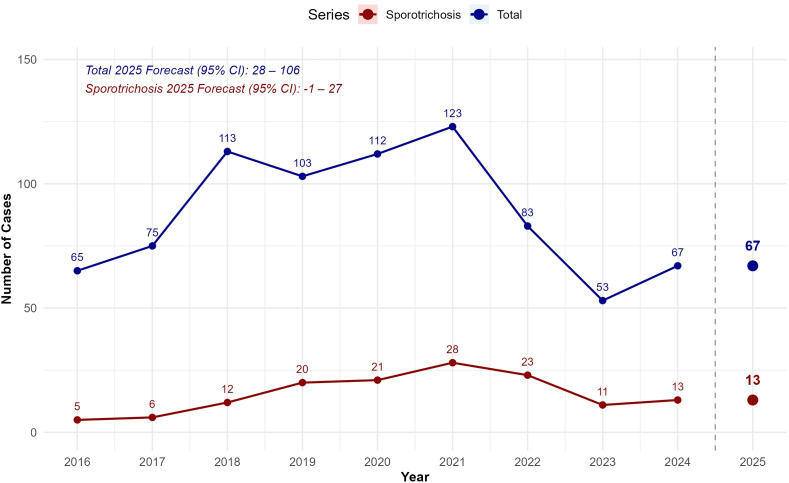
Estimate of total mycoses and sporotrichosis hospitalizations in Pernambuco, Brazil (2016-2025), including 95% Confidence Intervals (95% CI).

The annual number of hospitalizations for all mycoses from 2016 to 2024, along with a projection for 2025, is presented (Fig 5). Observed values increased from 65 in 2016–123 in 2021, fell to 53 in 2023, and rebounded to 67 in 2024. The model forecasts 67 admissions for 2025. The number of sporotrichosis hospital admissions was also traced (Fig 5), showing an increasing trend from five cases in 2016–28 in 2021, followed by a decrease and a slight rise in 2024. The model projected 13 admissions for 2025.

## Discussion

Our findings reveal a significant picture of opportunistic and endemic mycoses requiring hospitalization in PE, notably the abrupt increase in sporotrichosis admissions over nine years, with local incidence rates exceeding the national average. Aspergillosis, cryptococcosis, invasive candidiasis/candidemia, and histoplasmosis remained the most prevalent conditions both nationally and locally; however, sporotrichosis exhibited the most significant relative increase, underscoring its emergence as a public health concern in urban areas.

When comparing these data with other regional contexts in Brazil, notable variations were observed. A cross-sectional study conducted at a referral hospital in Belém (Pará State) between 2008 and 2017 reported 859 patients (2.5% of all admissions) identified candidiasis (41.2%) and cryptococcosis (36.1%) as the most frequent mycoses [21]. This difference highlights the highly localized nature of fungal epidemiology. Such variations may be attributed to specific ecological niches, distinct population demographic profiles, and regional healthcare practices or environmental factors that favor certain fungi. The analysis of annual incidence rates per 1,000 inhabitants reinforces this perspective. Although aspergillosis and cryptococcosis present the highest rates both in PE and nationwide, the state of PE exhibited higher incidence rates than the national average for chromoblastomycosis and sporotrichosis. This finding suggests a disproportionately high local burden of these specific infections, implying the influence of region-specific environmental conditions or exposure patterns that may contribute to their increased incidence.

The present study also recorded an overall mortality rate of 10.5% for hospitalizations due to mycoses in the state. Although this rate is lower than the 19.7% reported in the study by Ohnishi et al. [21], it underlines the inherent severity of these infections when they progress to the point of requiring hospitalization. This variation may be influenced by differences in data recording methodologies or in the attribution of outcomes within information systems such as SIH/SUS. Regardless of comparative figures, the mortality rate of 10.5% displays the serious nature of mycoses leading to hospitalization and the urgent need to enhance early diagnosis and antifungal therapies.

The findings also reveal a significant scenario of a sharp increase in sporotrichosis-related hospitalizations in PE, with local incidence rates surpassing the national average. The number of hospital admissions has risen from 5 cases in 2016–28 in 2021, followed by a slight decline in 2022–2023, reaching 13 cases in 2024. This trend in PE reflects a broader epidemiological shift in sporotrichosis across Brazil. Historically, the disease was considered rare and associated with specific occupational activities, with a geographically restricted distribution. Exposure was primarily environmental, linked to the saprophytic transmission route [22]. However, in recent decades, sporotrichosis has evolved into a widespread public health challenge, characterized by increasing zoonotic transmission, particularly in urban areas [12,23]. Cats are particularly susceptible to infection by *S. brasiliensis*, often developing severe forms of the disease characterized by a high fungal burden in their lesions. Their social behavior, which includes fighting and mutual grooming, facilitates the rapid dissemination of the fungus among individuals, resulting in both interspecific and intraspecific transmission [24].

The incidence of sporotrichosis in the Northeast region of Brazil has been increasingly reported [16,17,25]. Between 2016 and 2021, 1,176 feline cases were diagnosed in the state of PE, demonstrating significant zoonotic transmission and the overwhelming prevalence of *S. brasiliensis* as the etiological agent [17]. A substantial 163% increase in human cases was observed between 2017 and 2018, with patients receiving outpatient care in the state [16].

In comparison, the state of Rio de Janeiro (RJ) has experienced a long-term hyperendemic condition since 1998, with 14,490 human cases and 20,202 feline cases reported between 2011 and April 2023. Hospitalizations and deaths have shown a progressive increase over time [13,23]. The observation that isolates from PE are closely related to those from RJ, exhibiting low genetic differentiation, suggests a recent introduction of these strains [17]. Other emerging hotspots include Manaus, in the state of Amazonas, which has seen an exponential increase in both human (950) and animal (2,823) cases from 2020 to 2023, with cats implicated in 73.5% of human cases [12]. Neighboring states to PE, such as Rio Grande do Norte (122 human cases between October 2016 and December 2019, with 94.3% reporting contact with sick cats) and Paraíba, with reported cases of ocular sporotrichosis, also demonstrate the regional expansion of the disease [26,27].

The sociodemographic profile analysis of hospitalized patients in PE reveals that 57.3% of hospitalizations due to mycoses in general and 51.8% of hospitalizations due to sporotrichosis involved male individuals, with a mean age of 48 years and a high proportion of individuals self-identifying as mixed race (pardo), at 66%.These findings suggest a correlation between the burden of severe fungal infections and social vulnerability. Indicators such as low socioeconomic status, limited educational attainment, and non-white ethnicity are consistently associated with more severe cases linked to hospitalization and poorer outcomes [13,16,28]. Vulnerable populations often face barriers to early access to healthcare, live in conditions conducive to disease transmission (such as urban crowding and inadequate sanitation), and may exhibit a higher prevalence of comorbidities. The high proportion of “ethnicity not reported” in the study’s dataset reflects a systemic issue in public health records, which can obscure racial/ethnic disparities and hinder the accurate identification and targeted response to health inequities through specialized programs.

Regarding sex, although the study found a balanced distribution of hospitalizations due to sporotrichosis in PE (48.2% female vs. 51.8% male), this contrasts with observations from hyperendemic areas such as Rio de Janeiro, where women are more frequently affected due to close contact with sick cats in domestic settings. However, severe cases and fatalities are disproportionately observed in men, possibly due to overlap with HIV/AIDS endemicity [13,23,28,29]. In PE, Oliveira et. al [17] reported a predominance of male cases (70.8%) in overall human sporotrichosis between 2013 and 2022. This discrepancy between hospitalization data and general incidence may reflect differences in exposure patterns leading to hospitalization or variations in data sources.

The geographic distribution of hospitalizations for opportunistic and endemic mycoses in PE is heavily concentrated in Recife, while interior municipalities report far fewer admissions. Recent data revealed that transmission by cats was responsible for 99.4% of human cases in the state, indicating that the proliferation of the disease in the human population is intrinsically linked to the prevalence and management of the infection in the feline population. The spatial clustering in coastal and metropolitan municipalities reflects not only ecological factors, such as higher humidity and urban density, but also the role of domestic animal reservoirs. This urban clustering mirrors the zoonotic transmission of *S. brasiliensis* in densely populated settings [17]. Concurrently, sporotrichosis is spreading beyond its original urban hotspots into peripheral and rural zones [16,17]. Such a dual pattern, high-density epidemic hubs coupled with geographic expansion, indicates that control strategies must first deploy sustained, focused interventions in urban centers (e.g., mass cat sterilization and treatment programs alongside community education) and establish initiative-taking surveillance and rapid-response capacity in newly affected areas [30]. Decentralizing health services is crucial for containing existing hotspots and preventing the establishment of new endemic foci.

One noteworthy finding is that 46% of sporotrichosis hospitalizations were classified as unspecified forms (B42.9). This high proportion reveals critical data gaps that prevent precise epidemiological characterization and effective clinical management. It highlights systemic weaknesses in diagnostic reporting and in clinical training within the healthcare system. The lack of detailed clinical categorization hinders accurate assessment of disease spectrum and severity, thereby undermining targeted resource allocation and the implementation of effective public health interventions. Among cases with specified clinical forms, lymphocutaneous disease was the most prevalent (18.7%), followed by disseminated (17.3%), extracutaneous/other (13.7%), and pulmonary (4.3%).

The finding that nearly 20% of hospitalized sporotrichosis cases corresponded to the lymphocutaneous form raises questions regarding the criteria for hospital admission, given that this clinical presentation is classically managed on an outpatient basis. The rationale for hospitalizing clinically less severe forms is primarily logistical and structural, reflecting the healthcare environment in the state’s interior regions. Patients from remote areas with limited access to specialized health services often use the reference hospital as the primary gateway for infectious disease management. Regional primary health units sometimes lack specific training for the timely suspicion and diagnosis of sporotrichosis, resulting in patients being promptly referred to the hospital level, where specialized care (dermatology, infectology) is guaranteed, regardless of the initial severity of the condition. Additionally, the primary antifungals used for sporotrichosis include lipid formulations of amphotericin B for severe, disseminated, or systemic cases, and Itraconazole, which is employed for the treatment of lymphocutaneous and cutaneous forms, serving both as initial management for moderate cases and as step-down therapy following stabilization of more severe presentations.

Although the lymphocutaneous form predominates, the substantial share of systemic presentations is concerning, given their frequent association with increased severity and poorer outcomes. The rise in severe and atypical presentations of sporotrichosis has been linked to the heightened virulence of *S. brasiliensis* [23]. Reported cases of Sweet’s syndrome [31] and ocular sporotrichosis [11,27,32] underline the need for molecular identification to secure accurate diagnoses in complex scenarios. Reports of fatal pulmonary sporotrichosis [33,34] and osteoarticular involvement sequelae [35] have been increasingly reported, the last one affecting 12.2% of patients with bone sporotrichosis, including amputations (7.3%) and ankylosis (4.9%). Also, an increase in the number of cases, hospitalizations, and deaths of HIV-coinfected patients with sporotrichosis has been documented. In Rio de Janeiro, HIV coinfection was identified in 118 hospitalizations between 1999 and 2015. Notably, 25.3% of these hospitalizations and 27.3% of related deaths occurred between 2013 and 2015 [36,37]. It highlights the disease’s capacity to progress to severe, life-threatening forms, particularly in immunocompromised patients. Unfortunately, the SIH/SUS does not provide details of atypical cases or information on immunosuppression or other health conditions to help understand the reasons for current hospital admissions.

The temporal clustering of sporotrichosis admissions, particularly the surge observed between 2020 and 2022 (as shown in Fig 4), is hypothesized to be intrinsically linked to the socio-environmental shifts associated with the COVID-19 pandemic. Following the World Health Organization’s (WHO) declaration of COVID-19 as a pandemic in March 2020 and the implementation of global lockdowns, studies reported a 37% increase in cases and a rise in cat-transmitted sporotrichosis (CTS) clusters [11]. This sharp increase is hypothesized to be related to the higher rates of pet adoption, including cats and dogs, aimed at mitigating anxiety and depression caused by confinement. This pattern was observed across Brazil, where families incorporated pets into their homes to lessen anxiety among confined children during the SARS-CoV-2 outbreak. Given the established role of domestic cats as the primary reservoir and vector for the *S. brasiliensis* epidemic in Brazil, the intensified human-cat interaction within households during the pandemic likely accelerated the rate of zoonotic transmission in PE, providing a robust socio-epidemiological context for the observed surge in hospitalizations during this specific time window.

The mortality rate observed among sporotrichosis hospitalizations in PE was 5.8%, exceeding the national average of 3.4% reported in a systematic review [28]. This discrepancy could suggest that those patients may present with more advanced disease or a greater burden of underlying comorbidities predisposing them to severe outcomes. Or may therefore reflect delays in diagnosis or access to specialized care, reinforcing the urgent need to strengthen primary healthcare and the comprehensive management of comorbidities.

Currently, no single nationwide projection exists for future mycoses or sporotrichosis hospitalizations in Brazil. Our study’s estimates for 2025 in PE predict 67 hospitalizations for all mycoses and 13 for sporotrichosis, highlighting a sustained high burden on hospitals. These numbers highlight the endemic-epidemic nature of sporotrichosis and underscore the need for mandatory reporting, recording of transmission routes, and One Health interventions. It is especially relevant in PE, where human, animal, and environmental health intersect in complex ways. Effective control will require cross‑sectoral collaboration, integrating veterinary services, public health surveillance, and community education to interrupt transmission cycles.

Existing public health measures have not altered this trajectory, underscoring the need to shift from reactive responses to initiative-taking, long-term strategies with increased investment in prevention and primary care. Ongoing transmission, diagnostic delays, and treatment failures remain unchecked, suggesting that existing controls, while mitigating worse outcomes, are not breaking transmission chains or preventing severe disease. This persistence imposes chronic pressure on the health system and demands continuous allocation of resources. Policymakers must go beyond incremental adjustments and implement transformative, multisectoral approaches, strengthening epidemiological surveillance, enforcing compulsory notification, and ensuring detailed documentation of transmission routes and clinical presentations, to reduce the overall disease burden and its impact on hospital services.

This study is subject to limitations inherent to the use of secondary data. The main limitation is our dependence on the SIH/SUS database. Although this is a comprehensive source of hospitalization records, it lacks detailed clinical information, such as etiological confirmation by laboratory tests (culture or molecular), the specific clinical form of the disease (beyond broad ICD codes), and crucial individual clinical outcomes related to hospitalization. Most importantly, this lack of clinical specificity prevented us from reliably identifying proxies for host immunosuppression (e.g., HIV diagnosis, malignancy, or transplantation status) and the use of immunosuppressive drugs (e.g., corticosteroids). Consequently, the reported outcomes reflect raw hospitalization data without adjustment for patient vulnerability, a significant determinant of mycosis outcome.

Furthermore, the validity of any mycosis diagnosis depends on the correct notification and registration of ICD codes, which inevitably introduces classification and underreporting biases. The lack of access to reliable clinical details limits our ability to draw more robust inferences from the hospitalization data. Despite these inherent restrictions, the SIH/SUS database remains a vital surveillance tool for identifying epidemiological trends and highlighting the growing importance of opportunistic and endemic mycoses, particularly sporotrichosis, as a critical public health concern in PE.

## Conclusion

The present study provides compelling evidence of a critical public health challenge posed by the hospitalization incidence of opportunistic and endemic mycoses in Brazil, with prominence in the Brazilian northeastern state of Pernambuco. The rise in sporotrichosis cases, with the high admission burden and local occurrence rates surpassing the national average, emphasizes the urgent need for a transformative, multi-sectoral public health response. The concentration of cases in Recife, coupled with projections of a high and sustained hospital burden in 2025, highlights the critical need to strengthen epidemiological surveillance for this and other fungal infections. It is crucial to enforce compulsory notification effectively and ensure the SIH/SUS system records detailed information, including clinical forms and comorbidities. These measures are vital for enabling a better understanding of hospital admission cases and guiding more efficient control and targeted interventions for the population’s health.
